# Early Onset of Obesity and Adult Onset of Obesity as Factors Affecting Patient Characteristics Prior to Bariatric Surgery

**DOI:** 10.1007/s11695-018-3381-y

**Published:** 2018-07-18

**Authors:** Małgorzata Wrzosek, Klaudia Wiśniewska, Ada Sawicka, Marek Tałałaj, Grażyna Nowicka

**Affiliations:** 1 Department of Biochemistry and Pharmacogenomics, and Center for Preclinical Studies, Medical University of Warsaw, Banacha 1, 02-097 Warsaw, Poland; 2 Farmakon (students’ scientific group) at Department of Biochemistry and Pharmacogenomics, Medical University of Warsaw, Warsaw, Poland; 3 Centre of Promotion of Healthy Nutrition and Physical Activity, Institute of Food and Nutrition, Warsaw, Poland; 4 Department of Geriatrics, Internal Medicine and Metabolic Bone Diseases, Medical Centre of Postgraduate Education, Prof. W. Orlowski Hospital in Warsaw, Warsaw, Poland

**Keywords:** Obesity, Age at onset of obesity, Health complications

## Abstract

**Background:**

Patients who are slated for bariatric surgery vary in terms of their age at onset of obesity, duration of obesity, and their health complications. Therefore, we aimed to explore a relationship between the age at onset of obesity, metabolic parameters, and health problems in bariatric surgery candidates.

**Methods:**

A total of 469 unrelated adults with obesity prior to bariatric surgery were included in this study. The study group consisted of 246 individuals who became obese < 20 years of age, and 223 individuals who became obese ≥ 20 years. Clinical, biochemical, anthropometric assessments, and DXA-derived measures were taken.

**Results:**

Patients with early onset of obesity had a higher total body fat mass, and higher body fat percentage, and a 1.84 times higher risk of BMI above 40 kg/m^2^ than patients with adult onset of obesity (≥ 20 years). Multivariable logistic regression demonstrated that, among bariatric surgery candidates with early onset of obesity, the frequency of hypertension and type 2 diabetes was significantly lower than that in cases with an adult onset of obesity, despite a longer duration of obesity and higher BMI.

**Conclusions:**

The age at which an individual reaches obesity has a significant impact on patient characteristics on the day he or she is evaluated for bariatric surgery. A younger age at obesity onset is a predicting factor for a higher BMI in patients, but they are less likely to clinically manifest well-established consequences of obesity, such as diabetes or hypertension, compared to patients with adult onset of obesity.

## Introduction

Obesity is a complex chronic disease, characterized by an increase in body mass due to the growth of the boy’s fat tissues, leading to the development of numerous health complications. It is currently one of the world’s most serious health problems, affecting not only developed nations but developing nations as well. The worldwide incidence of obesity has risen to epidemic proportions [1], as indicated by the fact that, in 2016, over 650 million adults were obese. Particularly alarming are data showing that, over the past four decades, mean body mass indexes (BMIs) in children and adolescents aged 5–19 years have increased in most regions and countries, such that in 2016 the estimated number of girls worldwide who were obese reached 50 (24–89) million, and the number for boys reached 74 (39–125) million [2].

Patients with obesity are at risk of one or more of a great number of health problems, including type 2 diabetes, high blood pressure, heart disease, high total cholesterol and triglycerides, stroke, sleep apnea, and certain types of cancer [3–5]. However, not all obese patients have any of the metabolic disorders typically associated with excessive fat accumulation [6]. Depending on the diagnostic criteria adopted, the incidence of such cases is estimated at 10–30% [7–9]. Our earlier studies have shown that an older age at onset of obesity may be a significant factor determining the risk of developing weight-related diabetes [10]. Bariatric surgery is a recognized treatment method for severe obesity and is known to have superior outcomes compared to drug therapy for patients who have been unable to sustain weight loss by non-surgical means [11]. Patients applying for bariatric surgery vary with respect to age at onset of obesity and duration of obesity, and hence with respect to the severity of any obesity-related complications. Evaluating these differences is crucial in the approach to the candidate for bariatric surgery. This issue is therefore the subject of our further research, and the aim of this study was to assess differences in patient characteristics prior to bariatric surgery in relation to age at onset of obesity and the risk of obesity-related diseases, such as type 2 diabetes, hypertension, dyslipidemia, obstructive sleep apnea (OSA), and non-alcoholic fatty liver disease (NAFLD).

## Subjects and Methods

### Ethics Statement

The study was carried out in accordance with the principles of the Declaration of Helsinki. The whole study protocol and the consent procedure were approved by the Institutional Bioethics Committees (KB/127/2012 and KB/67/2017 at the Medical University of Warsaw; and 7/PB/2015 at the Medical Centre of Postgraduate Education). Written informed consent was obtained from each participant after a full explanation of the study.

### Patient Enrollment

A total of 469 unrelated individuals were enrolled in this study. All subjects were consecutively recruited on the basis of clinical investigation between September 2013 and September 2017 from patients who had been admitted to the Orlowski Hospital in Warsaw prior to bariatric surgery. All patients underwent standard pre-operative bariatric evaluation [12]. Inclusion criteria for bariatric surgery were a BMI above 40 kg/m^2^, or a BMI above 35 kg/m^2^ with at least one obesity-related morbidity, and failure of previous treatment of obesity [12, 13]. A detailed clinical history, including a history of obesity and a full physical examination, was obtained for each patient. In all subjects, anthropometric measurements (body weight, and height) were taken and BMI was calculated as the ratio of weight (kilograms) to the square of height (meters). Data on DXA-derived measures of total body fat (fat mass expressed as % fat mass and kg) was available for the whole group of participants. A person’s age at onset of obesity was ascertained by a physician prior to classification for bariatric surgery, based on a review of medical records in which previous body weight measures were reported. Out of 652 severely obese individuals who agreed to participate in this study and provided informed consent, we excluded 183 patients because of insufficient documentation for the age at onset of obesity, or for not fully completed medical data. The remaining 469 patients were included.

The age at onset of obesity was defined as either < 20 years (2- to 19-year-olds) or ≥ 20 (obesity in adults), as previously described [14, 15]. The duration of obesity was estimated by subtracting the current age from the age at onset of obesity.

### Analytical Procedures

Overnight peripheral fasting blood samples were taken from all subjects, and the serum was either isolated and used for analyses or stored at − 80 °C. All samples were analyzed by specialized clinical laboratory medical personnel. The laboratory analyses included measurements of total cholesterol, high-density lipoprotein cholesterol (HDL-C), low-density lipoprotein cholesterol (LDL-C), triglycerides, glucose, HbA1c (%), 25(OH)D, C-reactive protein (CRP), 25(OH)D, ASP, ALT, and erythrocyte sedimentation rate (ESR). Serum levels of interleukin 6 (IL-6) was determined by the ELISA method using the Diaclone Human IL-6 High Sensitivity ELISA kit.

### Diagnostic Criteria

Obesity was classified according to World Health Organization criteria [12], and subjects with BMI ≥ 30 kg/m^2^ were considered obese. Childhood obesity was diagnosed according to the International Obesity Task Force (IOTF) recommendations [16], based on the anthropometric data from the medical history.

Participants were classified as being hypertensive if they had an average blood pressure ≥ 140/90 mmHg, assessed as previously described [17], or by a previous diagnosis of hypertension and they were on hypertensive medication at the time of the interview. Patients were classified as diabetics based on the review of medical records (previous diagnosis of diabetes by a physician, and current use of glucose-lowering medications) and confirmed by current medical examination. The diagnosis was made using criteria consistent with those proposed by the American Diabetes Association [18] (an average fasting glucose concentration ≥ 126 mg/dl on two occasions, and/or 2 h glucose > 200 mg/dl during an oral-glucose-tolerance test, and/or a casual glucose > 200 mg/dl). Participants were classified as having dyslipidemia if they had received a diagnosis from a physician according to the National Cholesterol Education Program-Adult Treatment Panel III (ATP III) guidelines [19] and/or reported the use of lipid-lowering medications. Metabolic syndrome (MS) was defined according to the criteria of International Diabetes Federation (IDF) [20], which are as follows: central obesity defined as waist circumference ≥ 94 cm for men and ≥ 80 cm for women plus two or more of the following four factors: raised triglyceride level (≥ 150 mg/dl), reduced high-density lipoprotein (HDL) cholesterol level (for men < 40 mg/dl and for women < 50 mg/dl) or currently being treated for low HDL, raised blood pressure (systolic BP ≥ 130 mmHg or diastolic BP ≥ 85 mmHg, or treatment of previously diagnosed hypertension), raised fasting glucose concentration (≥ 100 mg/dl or previously diagnosed type 2 diabetes).

Patients were diagnosed with non-alcoholic fatty liver disease (NAFLD) based on liver ultrasonography (USG) findings. A hepatic USG examination was performed by experienced radiologists. In the assessment, NAFLD was defined as diffuse hyperechogenicity of the liver relative to the kidneys, ultrasound beam attenuation, and poor visualization of intrahepatic structures [21]. All participants underwent standard overnight assessment (polysomnography, PSG) to evaluate the presence of OSA. OSA was assessed by the Apnea–Hypopnea Index (AHI), which is the number of complete (apneas) or incomplete (hypopneas) obstructive events per hour of sleep, and defined as AHI ≥ 5 [22].

### Statistical Analysis

Data was analyzed with the Statistica 12.0 program. Categorical variables were described with number (percentage) and analyzed by chi-square test. The concordance with normal distribution for continuous variables was calculated with the Shapiro-Wilk and Kolmogorov–Smirnov tests. Continuous variables were described with a median (interquartile range) for non-normal data. We grouped participants into two categories: the first consisted of patients with an early onset of obesity (before the age of 20 years) and the second with an adult onset of obesity (individuals who developed obesity after 20 years of age). In addition, to compare patients with early and adult onset of obesity within a more narrow overall age range, we divided the subjects into quartiles (Q1–Q4) based on their age. Mann–Whitney rank tests were used to assess differences between groups with age of obesity onset < 20 years and with age of obesity onset ≥ 20 years. Multiple logistic regression was used to identify factors associated with early onset of obesity. Variables that were significantly related with age of obesity onset in univariate analyses were then included in the multivariate logistic regression model. Qualitative variables were coded as 0–1 dummy variables. Results from the logistic regression model are presented as odds ratios (OR) with 95% confidence interval (CI). In all analyses, a *p* value < 0.05 was considered statistically significant.

## Results

Clinical and biochemical characteristics of study participants (*n* = 469) are summarized in Table 1. Altogether, participating in this study were 246 individuals who had been obese since before the age of 20 years, whose median age at onset of obesity was 7.0 (IQR 6.0–10.0) years, and 223 individuals who had become obese at age 20 or older, whose median age at onset of obesity was 29.0 (IQR 24.0–40.0) years.

**Table 1:** Clinical and biochemical characteristics of the study participants in relation to age at onset of obesity

Variables	The age at onset of obesity		*p value
	< 20 years *n* = 246	≥ 20 years *n* = 223	
Age in years	37.0 [32.0–45.0]	49.0 [41.0–57.0]	*0.001*
Age at onset of obesity (years)	7.0 [6.0–10.0]	29.0 [24.0–40.0]	*< 0.001*
Duration of obesity (years)	29.5 [21.0–38.0]	16.0 [10.0–22.0]	*< 0.001*
Weight (kg)	121.5 [107.5–135.0]	113.0 [100.0–131.0]	*< 0.001*
Height (cm)	168.5 [163.0–174.0]	168.0 [161.5–175.0]	0.502
BMI	42.0 [39.0–46.0]	40.0 [36.0–45.0]	*0.001*
Fat %	45.0 [41.0–48.0]	43.0 [37.0–47.0]	*< 0.001*
Fat mass (kg)	51.0 [44.0–58.0]	46.0 [39.0–53.0]	*< 0.001*
Systolic blood pressure (mmHg)	133.0 [120.0–143.0]	129.0 [119.0–142.0]	0.250
Diastolic blood pressure (mmHg)	75.0 [70.5–81.0]	75.0 [70.0–81.0]	0.800
Fasting glucose (mg/dl)	100.0 [85.0–103.0]	115.0 [94.0–124.0]	*< 0.001*
HbA1c (%)	5.7 [5.4–6.2]	6.1 [5.6–6.8]	*< 0.001*
Total cholesterol (mg/dl)	186.0 [157.0–209.0]	178.0 [152.0–213.0]	0.295
LDL cholesterol (mg/dl)	114.0 [88.0–132.0]	106.0 [78.0–138.0]	0.083
HDL cholesterol (mg/dl)	41.0 [35.0–49.0]	41.0 [35.0–49.0]	0.883
Triglycerides (mg/dl)	137.0 [99.0–181.0]	142.0 [101.0–188.0]	0.363
Alanine aminotransferase (U/l)	24.0 [32.0–59.0]	24.0 [35.0–58.0]	0.176
Aspartate aminotransferase (U/l)	43.0 [19.0–59.0]	44.5 [19.0–58.0]	0.596
25(OH)D (ng/ml)	17.0 [11.0–22.6]	16.0 [11.0–21.0]	0.390
Apnea–Hypopnea Index	4.3 [1.2–13.9]	6.3 [2.9–19.1]	*0.003*
IL-6 (pg/ml)	2.5 [1.5–4.3]	2.6 [1.5–4.1]	0.724
ESR	15.0 [8.5–24.0]	15.0 [8.0–25.0]	0.665
CRP (g/l)	6.7 [3.1–12.6]	5.6 [2.9–11.0]	0.128

Values are given as median (interquartile range)

*Abbreviations*: *BMI*, body mass index; *CRE*, creatinine; *HbA1c*, glycosylated hemoglobin; *HDL cholesterol*, high-density lipoprotein cholesterol; *LDL cholesterol*, low-density lipoprotein cholesterol; *ESR*, erythrocyte sedimentation rate; *CRP*, C-reactive protein

*Mann–Whitney tests, *p* value < 0.05 are in italics

We found significant differences in terms of obesity duration (years), weight, BMI, body fat percentage, body fat mass, fasting glucose, HbA1C, and AHI between the two groups (Table 1). The study group with an early onset of obesity (< 20 years) had longer duration of obesity than participants with adult onset of obesity (median 29.5 versus 16.0 years). An earlier onset of obesity (< 20 years) was associated with higher BMI, higher total body fat (expressed as fat % and fat mass kg, Table 1), and a 1.84 times greater risk (OR = 1.84, 95% CI 2.37–4.52) of BMI above 40 kg/m^2^ (Table 2), despite the fact that candidates with an early onset of obesity were younger on the day they were qualified for bariatric surgery than candidates with adult onset of obesity.

**Table 2:** Results of a logistic regression determining factors associated with early onset of obesity (< 20 years) in bariatric surgery candidates

Variables	The age at onset of obesity		Crude OR (95% CI)	*p* ^**a**^	Adjusted OR (95% CI)	*p* ^**b**^
	< 20 years *n* = 246	≥ 20 years *n* = 223				
Diabetes, *n* (%)						
Yes No	35 (14.0) 211 (86.0)	91 (41.0) 132 (59.0)	*0.24 (0.15–0.38)*	*< 0.001*	*0.58 (0.38–0.83)*	*0.017*
Hypertension, *n* (%)						
Yes No	131 (53.0) 115 (47.0)	173 (78.0) 50 (22.0)	*0.33 (0.22–0.49)*	*< 0.001*	*0.47 (0.27–0.82)*	*0.008*
Dyslipidemia, *n* (%)						
Yes No	100 (41.0) 146 (59.0)	129 (58.0) 94 (42.0)	*0.50 (0.34–0.72)*	*< 0.001*	0.77 (0.47–1.24)	0.282
MS, *n* (%)						
Yes No	124 (50.0) 122 (50.0)	167 (75.0) 56 (25.0)	*0.34 (0.23–0.50)*	*< 0.001*	0.96 (0.51–1.80)	0.894
BMI, *n* (%)						
Above 40 30–39.9	166 (67.0) 80 (33.0)	126 (56.5) 97 (43.5)	*1.60 (1.10–2.33)*	*0.014*	*1.84 (1.15–2.92)*	*0.009*
NAFLD, *n* (%)						
Yes No	191 (78.0) 55 (22.0)	193 (87.0) 30 (13.0)	*0.54 (0.33–0.88)*	*0.012*	0.78 (0.43–1.42)	0.433
Sex, *n* (%)						
Male Female	51 (21.0) 195 (79.0)	79 (35.0) 144 (65.0)	*0.48 (0.32–0.72)*	*< 0.001*	0.78 (0.47–1.29)	0.333
OSA, *n* (%)						
Yes No	117 (48.0) 129 (52.0)	129 (58.0) 94 (42.0)	*0.66 (0.49–0.95)*	*0.026*	0.79 (0.49–1.26)	0.318

*p* value < 0.05 are in italics

*MS*, metabolic syndrome; *NAFLD*, non-alcoholic fatty liver disease; *OSA* obstructive sleep apnea; *OR*, odds ratio; *CI*, confidence interval

^a^Crude logistic regression analysis

^b^Adjusted logistic regression analysis (after controlling for all other significant factors)

Among the participants whose age at obesity onset was < 20 years, 14% had type 2 diabetes, as opposed to 41% of those whose age at obesity onset was ≥ 20 years (*p* < 0.001). Thus, significantly lower serum glucose and HbA1c% was recognized (Table 1) and the frequency of type 2 diabetes was significantly lower (OR = 0.24; 95% CI = 0.15–0.38) in the former group than in the latter (Table 2).

The prevalence of hypertension and dyslipidemia was significantly lower among subjects who had been younger at onset of obesity (< 20 years) than in those who had been older at onset of obesity (≥ 20 years) (Table 2). Considering that hypertension, type 2 diabetes, and dyslipidemia are components of metabolic syndrome (MS), the frequency of MS was significantly lower in patients with early onset of obesity than in patients with adult onset of obesity (50.0 versus 75.0%, *p* < 0.001).

A lower median Apnea–Hypopnea Index (Table 1) and lower proportion of individuals with OSA were recognized among the participants who had been younger at onset of obesity (48%) compared to the participants who had been older (58%, *p* < 0.001, Table 2). Likewise, the prevalence of NAFLD was significantly lower in those with early onset (< 20 years) than in the patients with adult onset of obesity (78.0 versus 87.0%, *p* < 0.001, Table 2).

Variables that were significant in univariable models (i.e., type 2 diabetes, hypertension, dyslipidemia, MS, BMI above 40, NAFLD, gender, OSA) were then combined in the multivariable logistic regression model. The regression demonstrated that, among bariatric surgery candidates who became obese at < 20 years, the frequency of hypertension (OR = 0.47, 95% CI 0.27–0.82) and type 2 diabetes (OR = 0.58, 95% CI 0.38–0.83) was significantly lower, despite a higher prevalence of BMI above 40, than in patients who had become obese at ≥ 20 years (Table 2).

Median age of the group was 43 years, the 25th and 75th percentiles for age were 35 and 53 years, respectively. For comparing patients with early and adult onset of obesity, who are at the same age, we divided the subjects into quartiles (Q1–Q4), with each quartile containing 25% of the study population from the youngest age to the oldest, as shown in Table 3. In Q4, 23% (*n* = 27) of subjects had an early onset of obesity (< 20 years), while 81% (*n* = 100) in Q1, 59% (*n* = 72) in Q2, and 44% (*n* = 47) in Q3 had an early onset of obesity (Table 3).

**Table 3:** Characteristics of the bariatric surgery candidates, divided into four quartiles (Q1–Q4) from the youngest to the oldest age, in relation to age at onset of obesity

	Q1 (*n* = 123) M = 31 years; range 18–35		Q2 (*n* = 123) M = 39 years; range 36–43		Q3 (*n* = 107) M = 48 years; range 44–52		Q4 (*n* = 116) M = 58 years; range 53–67	
Onset of obesity	< 20 years	≥ 20 years	< 20 years	≥ 20 years	< 20 years	≥ 20 years	< 20 years	≥ 20 years
*n* (%)	100 (81%)	23 (19%)	72 (59%)	51 (41%)	47 (44%)	60 (56%)	27 (23%)	89 (77%)
Males (%)	19%	17%	22%	35%	23%	28%	19%	46%
Duration of obesity (years)	20 (14–25)	9 (7–11)	30 (26–34)	15 (10–19)	41 (37–45)	20 (15–22)	51 (47–55)	20 (12–27)
Age at onset of obesity (years)	7 (6–12)	24 (22–25)	7 (5–13)	25 (22–30)	7 (5–6)	29 (25–34)	7 (2–7)	40 (32–46)
BMI	42 (38–45)	40 (37–44)	42 (39–47)	42 (38–47)	44 (40–49)	41 (36–47)	41 (37–44)	40 (36–45)
Fat %	44 (41–48)	43 (40–48)	45 (42–48)	43 (39–46)	46 (41–48)	43 (37–47)	43 (39–46)	41 (35–47)
Fat mass (kg)	52 (44–58)	48 (43–55)	51 (43–58)	46 (39–52)	54 (45–60)	47 (40–57)	45 (38–48)	44 (36–50)
Diabetes (%)	6%	17%	11%	20%	23%	35%	37%	63%
Hypertension (%)	30%	35%	58%	75%	72%	77%	93%	91%
BMI above 40 (%)	69%	57%	65%	61%	74%	55%	55%	55%

Values are given as median (interquartile range) or *n* (%)

In Q3, the risk of having a BMI above 40 was significantly higher in patients with early onset of obesity than in patients who had become obese at ≥ 20 years (OR = 2.39, 95% CI 1.03–5.52; Table 4) and in Q3 similar percentages of patients with early and adult onset of obesity were recognized.

**Table 4:** Results of a logistic regression determining factors associated with early onset of obesity (< 20 years) in bariatric surgery candidates divided into four quartiles (Q1–Q4) from the youngest to the oldest age

	Q1 (*n* = 123)			Q2 (*n* = 123)			Q3 (*n* = 107)			Q4 (*n* = 116)		
Early age of obesity onset	Odds ratio	95% CI	*p*	Odds ratio	95% CI	*p*	Odds ratio	95% CI	*p*	Odds ratio	95% CI	*p*
Diabetes	0.30	0.07–1.19	0.078	0.84	0.53–1.34	0.458	0.74	0.44–1.23	0.244	*0.46*	*0.22–0.96*	*0.038*
Hypertension	0.80	0.31–2.21	0.654	0.48	0.22–1.06	0.066	0.79	0.33–1.93	0.609	1.23	0.24–6.30	0.797
BMI above 40	1.71	0.67–4.34	0.259	1.21	0.57–2.57	0.610	*2.39*	*1.03–5.52*	*0.039*	1.12	0.45–2.75	0.808

Adjusted logistic regression analysis (after controlling for all other significant factors). *p* value < 0.05 are in italics

*OR*, odds ratio; *CI*, confidence interval

The longest median obesity duration among bariatric surgery candidates who became obese at < 20 years was found in Q4 (51 years), but the frequency of type 2 diabetes (OR = 0.46, 95% CI 0.22–0.96) was significantly lower than that in patients who had become obese at ≥ 20 years (Tables 3 and 4). In other quartiles, the lower frequency of type 2 diabetes among patients with early-onset obesity is also indicated (Table 3).

## Discussion

Obesity among both children and adults is a serious public health challenge around the world [23]. Data available in published literature demonstrates that (1) obese adolescents were significantly more likely to develop severe obesity in young adulthood than normal-weight or overweight adolescents [14], (2) around 55% of obese children will go on to be obese in adolescence, and (3) around 80% of obese adolescents will still be obese in adulthood [24].

In the present study, we demonstrated that an earlier obesity onset (< 20 years), compared to later onset, was associated with greater weight and BMI. Bariatric surgery candidates with obesity onset < 20 years had higher total body fat mass and fat percentage and a 1.84 times higher risk of BMI above 40 kg/m^2^ than those with adult-onset obesity. These results concur with long-term serial data from the Fels Longitudinal Study [25], which showed that both males and females whose BMI surpassed 25 earlier in life had significantly greater weight and BMI than those with late onset of overweight. A higher BMI during childhood and adolescence is associated with an increased risk of the health consequences of obesity, such as type 2 diabetes, hypertension, dyslipidemia, carotid-artery atherosclerosis, elevated low-density lipoprotein cholesterol levels, reduced high-density lipoprotein cholesterol levels, and elevated triglyceride levels in adulthood [26, 27], which in consequence increased cardiovascular risk [27–29]. In addition, the Kaunas Cardiovascular Risk Cohort study, whose participants were 12–13 years old at the time of the baseline survey, showed in the 35-year follow-up survey (*n* = 506) that childhood BMI was predictive for metabolic syndrome, hyperglycemia or type 2 diabetes, and elevated level of high-sensitivity CRP in adulthood. However, no relationship was found between childhood anthropometric measurements, arterial hypertension, a raised level of triglycerides, or a reduced level of HDL cholesterol [30]. This suggests that the relationship between early age of obesity and the risk of metabolic disorders in the future is a complex relationship, which results directly from the fact that obese people are not homogeneous in terms of metabolic profile [31].

Our data demonstrated that, among bariatric surgery candidates whose age at onset of obesity was < 20 years, the frequency of hypertension and type 2 diabetes was lower than that in cases with adult-onset obesity, despite being obese for longer and having higher BMIs. This suggests that the age at which obesity began may determine the picture of metabolic disorders present in obese patients qualified for bariatric surgery. Moreover, Goday et al. demonstrated that overweight and obese patients who were metabolically healthy tended to be younger and were more likely to be female and/or to participate in physical exercise [32]. It should therefore be emphasized that a person’s age is an important risk factor for metabolic disorders, which is why the prevalence of metabolic consequences in our study was lower in the patients who had developed obesity earlier in life. Moreover, in previous studies, a low prevalence of glucose intolerance was seen in children and adolescents of European origin aged 6–18 years even if markedly obese [33]. In addition, Brochu et al. showed that early onset of obesity (< 20 years of age) was associated with a more favorable metabolic profile and with a lower accumulation of visceral adipose tissue in metabolically healthy but obese women [34]. Accordingly, the age of the patient may determine how fat tissue is distributed. Early onset of obesity may be associated with less visceral fat accumulation and less liver fat, which influence the development of complications connected with obesity regardless of the total amount of body fat. Such a body fat distribution profile may persist along lifespan and result in lower prevalence of diabetes in patients who develop obesity earlier in life as observed in our study.

Obesity is also a significant factor for an increased risk of OSA, or the worsening of symptoms, as measured by the average number of apnea and hypopnea episodes per hour of sleep (Apnea–Hypopnea Index, AHI). Furthermore, the condition is more common with age [35]. Observational studies show that reducing body mass, whether by proper diet or surgical procedure, significantly reduces AHI and can facilitate the treatment of OSA [36, 37]. This points attention to the fact that weight loss, especially in young people, can effectively decrease the risk of obstructive sleep apnea [38, 39]. In the case of the patients in our study, we observed that those who had become obese at a more advanced age showed significantly higher AHI than those with an early onset of obesity (< 20 years of age). It should be emphasized that implementing effective long-term weight loss strategies early can help reduce the risk of developing obstructive sleep apnea for this group of patients.

Meanwhile, we are aware that the present study has certain limitations. No evaluations were carried out regarding the dietary assessment of those qualified for the study, and these factors no doubt play an immense role in reducing the risk of developing metabolic disorders. Another factor not considered in the present study is the amount of visceral adipose tissue, which can be linked with a higher risk of metabolic disorders [40]. However, we did evaluate the indicators of inflammation, such as IL 6 and CRP, for which we did not observe statistically significant changes between groups, indicating that an early age of obesity development and its longer duration did not worsen a systemic inflammation.

Our observations suggest that a person’s age at onset of obesity should be a factor that is considered when evaluating a patient for bariatric surgery. In contrast to the current opinion that a longer period of obesity is connected with a greater number of health complications, we have shown that in patients who were of younger age at onset of obesity, despite their long duration of obesity, the risk of metabolic disorders was still lower than that in patients in similar age who developed obesity after 20 years of age. Therefore, it can be suggested that, in patients with an earlier onset of obesity, expedient decisions about bariatric surgery could prevent the development of age-related obesity complications.

It seems necessary to conduct further research in this area and to determine whether an early age of obesity can indeed be related to metabolically healthy obesity (MHO). Determining this dependence would be of great importance in the case of evaluating patients for surgery, because the available data indicate that people with MHO, compared to obese individuals who have metabolic disorders, may not benefit significantly from lifestyle changes and dietary approaches [41, 42].

In conclusion, in patients with early-onset obesity, the rate of development of metabolic diseases is slower despite the longer duration of obesity. Awareness of this fact is important for the qualification process for bariatric surgery, and a lack of metabolic disturbances should not be a factor delaying surgical intervention, because qualifying these patients for bariatric surgery earlier can prevent the appearance of such disorders.
